# Psychosocial working characteristics before retirement and depressive symptoms across the retirement transition: a longitudinal latent class analysis

**DOI:** 10.5271/sjweh.3889

**Published:** 2020-09-01

**Authors:** Julia K Åhlin, Paraskevi Peristera, Hugo Westerlund, Linda L Magnusson Hanson

**Affiliations:** 1 The Division for Epidemiology, Stress Research Institute, Department of Psychology, Stockholm University, Stockholm, Sweden

**Keywords:** depression, effort–reward imbalance, job control, job demand, job strain, longitudinal study, mental health, older worker, SLOSH, stress, Swedish Longitudinal Occupational Survey of Health, work stress

## Abstract

**Objectives:**

Retirement is a major life transition. However, previous evidence on its mental health effects has been inconclusive. Whether retirement is desirable or not may depend on pre-retirement work characteristics. We investigated trajectories of depressive symptoms across retirement and how a number of psychosocial working characteristics influenced these trajectories.

**Methods:**

We included 1735 respondents from the Swedish Longitudinal Occupational Survey of Health (SLOSH), retiring during 2008–2016 (mean retirement age 66 years). They had completed biennial questionnaires reporting job demands, decision authority, workplace social support, efforts, rewards, procedural justice and depressive symptoms. We applied group-based trajectory modelling to model trajectories of depressive symptoms across retirement. Multinomial logistic regression analyses estimated the associations between ­psychosocial working characteristics and depressive symptom trajectories.

**Results:**

We identified five depression trajectories. In four of them, depressive symptoms decreased slightly around retirement. In one, the symptom level was initially high, then decreased markedly across retirement. Perceptions of job demands, job strain, workplace social support, rewards, effort–reward imbalance and procedural justice were associated with the trajectories, while perceptions of decision authority and work efforts were only partly related to the trajectories.

**Conclusions:**

We observed a rather positive development of depressive symptoms across retirement in a sample of Swedish retirees. For a small group with poor psychosocial working characteristics, symptoms clearly decreased, which may indicate that a relief from poor working characteristics is associated with an improvement for some retirees. However, for other retirees poor working characteristics were associated with persistent symptoms, suggesting a long-term effect of these work stressors.

With increasing life expectancy in developed countries and population aging, many governments move towards increased retirement ages (1), stressing the importance of health promotion for healthy and active aging. However, the timing of retirement has been debated (2). Retirement itself is a major life transition and may be important for maintained well-being. However, previous research regarding mental health across retirement has been inconclusive. Beneficial effects of retirement have been observed (3, 4). For example, purchases of anti­depressants have been found to decrease after retirement (5). Others have found unchanged purchases of psychotropic drugs/antidepressants, and self-reported depress­ive symptoms following retirement (6–8). Conversely, retirement has also been associated with increasing depressive symptoms (9). As depression in older adults is a major public health concern – associated with large costs in terms of premature mortality, morbidity and lower quality of life (10) – a greater understanding of mental health across retirement is important for identifying effective intervention strategies.

It has been suggested that retirees’ well-being across retirement does not follow a uniform pattern, (11, 12) which may explain the heterogeneity in findings. The association between retirement and health likely depends on the reasons for retirement, eg, statutory and early voluntary retirement has been found to be associated with improved health, while the findings for retirement due to ill-health have been the opposite, suggesting a health selection into retirement (4). Whether retirement is desirable or not can also depend on work characteristics (13–16). It is well known that poor work characteristics could affect subsequent mental health negatively (17), but it is uncertain whether there are long-term effects. Very few studies have investigated the role of (psychosocial) working conditions in relation to mental health or depression following retirement (16, 18, 19). One study found that poor psychosocial work environment in midlife was associated with higher depressive symptoms during retirement, suggesting chronic effects of work stress on mental health (18). On the other hand, relief from work-related stress/strain has been suggested to explain why retirement affects health (20). A poor work environment before retirement has been associated with higher prevalence of suboptimum health while in work, but a greater retirement-related improvement in health (13). A recent study found that individuals with more disadvantageous working conditions experienced more substantial improvements in mental health following retirement, especially in the short term (16).

In the present study, we investigate trajectories of depressive symptoms across retirement, and how a number of psychosocial working characteristics in the end of working life may influence these trajectories. This study adds to the rather inconsistent literature regarding mental health across the transition from work to retirement, by considering depressive symptoms specifically and that symptoms may develop differently over time for different groups of individuals. In addition, this study investigates the role of a number of specific psychosocial working characteristics in depressive symptom development across the retirement transition, some of which have not been examined in the previous literature on mental health across retirement.

## Methods

### Data and study population

We used data from the Swedish Longitudinal Occupational Survey of Health (SLOSH), a cohort survey of individuals aged 16–64 years from across the entire country and fairly representative of the Swedish working population (21). SLOSH participants have been followed-up by postal self-completion questionnaires biennially, since 2006 until 2018 (waves 1–7) thus far, with response rates of 48–65%. Some participants have been followed up since 2006, while others have been followed up since 2008, 2010, or 2014. One version of the questionnaire is for people in paid work, defined as gainful employment of ≥30% of full-time on average during the past three months (‘workers’), and another version for people working less, or who have left the labor force temporarily or permanently (‘non-workers’). More details of the SLOSH study can be found elsewhere (21).

### Inclusion and exclusion criteria

**Figure 1:**
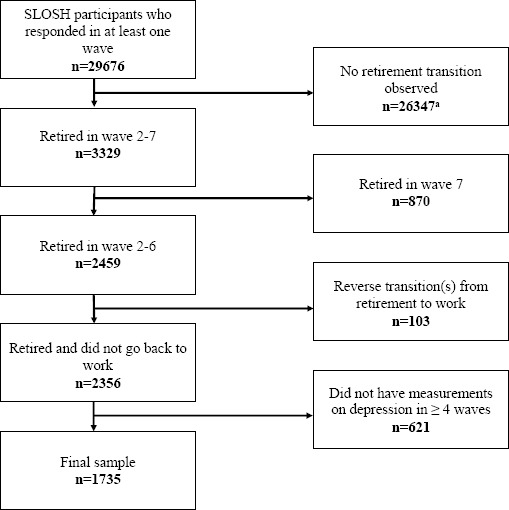
Flowchart illustrating the sample selection from SLOSH 2006–2018. ^a^ Among the 26 347 participants who were excluded, some individuals did not respond in ≥2 waves, some individuals remained working, and some made transitions from or to work/non-work other than retirement.

The present study is based on all currently available waves (1–7) in SLOSH. In total, 29 676 individuals (73% of the total cohort) responded to at least one survey in 2006–2018. We selected only participants for whom we could observe a retirement transition, defined as going from paid work in one wave (completed the questionnaire for ‘workers’) to being retired (completed a questionnaire for ‘non-workers’ and reported being retired) in the following wave during waves 2–6. Individuals were classified as retired if they reported being old age retirees or receiving another type of pension (eg, contractual pension) on a full-time basis. Retirement due to ill-health (like disability pension, or early retirement on health grounds) was not classified as retirement since health trajectories are likely to differ from those of individuals going through old age retirement/other type (12). We excluded participants who reported transitions from retirement back to paid work, so-called unretirement (N=103), and 621 individuals who had not reported depressive symptoms in ≥4 waves. Thus, our sample included 1735 individuals (figure 1). A sensitivity analysis of the 621 individuals compared to the 1735 included individuals is presented in the supplementary material (www.sjweh.fi/show_abstract.php?abstract_id=3889) table S1 and showed no statistically significant differences between the excluded and included individuals. Among those included, 31% had data on depressive symptoms in seven waves, 41% in six waves, 17% in five waves, and 11% in four waves. The majority had data on depressive symptoms both before and after retirement (N=1639).

### Psychosocial working characteristics

We included a number of psychosocial working characteristics from several dominating theoretical work stress models in waves 1–6 assessed by self-reports. Job demands, job control and workplace social support were measured by the Demand–Control–Support Questionnaire (22, 23). We specifically analyzed the subdimension decision authority of job control, since the subdimension skill discretion may be of less relevance in the modern working life. We used median split for classifying high and low demands, decision authority and social support. In addition, a variable combining demands and control was created according to the Job Strain Model (24). High demands and low control was defined as job strain, while the other three combinations were defined as no job strain.

Moreover, we used the short version of the effort–reward imbalance (ERI) questionnaire, which has shown to have satisfactory psychometric properties (25–27), to assess work efforts and rewards. Median split was used to classify high/low efforts and rewards, in order to facilitate the interpretation and comparison of the exposure variables. We also calculated the ERI ratio, where a ratio >1 was classified as ERI.

Procedural justice was measured with a seven-item scale (28). Median split was used to classify high/low procedural justice.

We assessed the following covariates as potential confounders: sex, age, civil status, occupational position, physical inactivity, excessive alcohol consumption, smoking, cardiovascular disease and diabetes, in line with similar studies (18, 19). The working characteristics and covariates were derived from the wave prior to reporting retirement, but if the variable was missing in that wave, data from two waves prior to retirement was used. A detailed description of the exposure and covariate variables and depressive symptoms is available in the supplementary material.

### Depressive symptoms

Depressive symptoms were assessed in waves 1–7 using the subscale Symptom Checklist-core depression (SCL-CD_6_) (29) of the (Hopkins) Symptom Checklist (SCL-90) (30). Participants reported on a five-point Likert scale to what extent during the last week they had experienced: feeling blue, feeling no interest in things, feeling lethargic or low in energy, worrying too much about things, blaming oneself for things, and feeling everything is an effort. We used a sum scale serving as an indicator of depressive symptoms severity, ranging from 0–24. A score between 0–6 has been suggested to indicate no depression, 7–9 doubtful depression, 10–11 mild depression, 12–15 moderate depression and 16–24 severe depression, similar to the ICD-10 diagnostic system (31).

### Statistical analyses

First, we conducted descriptive analyses to investigate how the level of depressive symptoms changed across retirement. Second, we applied group-based trajectory modelling (GBTM) to model trajectories of depressive symptoms across retirement using the plugin STATA TRAJ (32). GBTM identifies subgroups of individuals who follow a similar developmental course over time or age, in terms of a repeatedly measured behavior or phenomena (33). Time was years before and after retirement, ranging from nine years (corresponding to five waves) before retirement, to eleven years (corresponding to six waves) after retirement. The first wave a participant reported being retired was assigned +1 year, as the retirement transition took place sometime between years -1 to +1.

To decide the optimal number of trajectory groups and their complexity level (ie, the polynomial shape) that best described the trajectories, we followed the main principles as described more in detail previously (33–35). Briefly, we proceed by comparing lower number of trajectory groups to higher, after also identifying the most appropriate shape of the trajectories in that group (starting from cubic to linear). The models were compared through model fit using Bayesian Information Criterion (BIC) (36, 37) with lower BIC indicating a better fitting model. However, BIC can sometimes continue to decrease as more trajectory groups are added (33). Therefore, we considered a model with more groups (and thus lower BIC) inferior than a model with less groups, if a trajectory group in that larger model contained <1% of the sample, when the model no longer captured new distinctive features of the data, or when entropy (index of classification accuracy ranging from 0–1 with values closer to 1 indicating better precision) (38), or average posterior probabilities of assignment (APPA; preferably >0.7) (33) declined. We assumed a censored normal distribution (39).

Once the optimal trajectory model for depressive symptoms was identified, we investigated the distribution of pre-retirement characteristics and psychosocial working characteristics in the trajectory groups. Then, we examined how pre-retirement levels of job demands, decision authority, job strain, workplace social support, efforts, rewards, ERI and procedural justice were associated with membership in the depression trajectory groups by fitting multinomial logistic regression models. First, crude models were fitted for each predictor separately. Second, the models were adjusted for sex, age, civil status and occupational position pre-retirement. Third, the models were additionally adjusted for physical inactivity, excessive alcohol consumption, smoking, cardiovascular disease and diabetes.

## Results

### Average depressive symptoms

**Figure 2:**
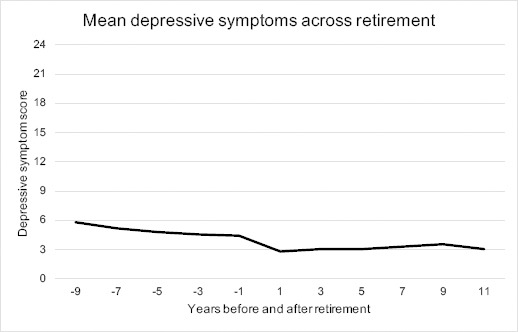
Mean depressive symptoms across retirement measured by the Symptom Checklist-core depression (SCL-CD6) among 1735 SLOSH participants between 2006 and 2018.

When investigating the mean scores of depressive symptoms across retirement, the mean symptom level was 5.9 [standard deviation (SD) 5.1] nine years before retirement and successively decreased until retirement. Symptoms were lowest the first time point after retirement (mean 2.9, SD 3.8) (see figure 2). The mean change in depressive symptom scores between the wave before and the wave individuals reported being retired was -1.5, indicating a reduction in symptoms. Symptoms decreased in 53.5% of the sample, while it remained unchanged in 23.7% and increased in 22.9% when comparing the wave before with the wave of retirement.

### Trajectories of depressive symptoms across retirement

**Figure 3:**
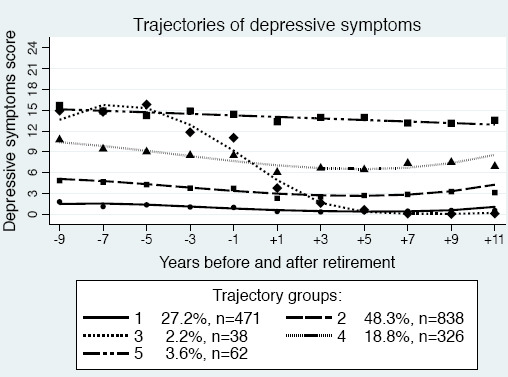
Trajectories of depressive symptom scores (0–24) across retirement in the SLOSH study (N=1735) and the proportion of retirees in each group. Trajectory group labels: 1=no depression, stable (very low), 2=no depression, stable (low), 3=moderate depression, considerably decreasing, 4=mild depression, decreasing, 5=moderate depression, stable.

To assess different patterns of depressive symptoms across retirement in the study population, we tested trajectory models with up to seven trajectory groups. However, we considered the five-group model as the best because this model provided new distinctive features of the data compared to four groups, entropy was second best (0.82), APPA was satisfactory (0.88), and both entropy and APPA decreased when adding a sixth group. The five trajectories were labelled according to symptom level at baseline and stability/change across the retirement (figure 3). Group 1 – *no depression, stable (very low)* (N=471) – symptoms were very low across the time period, indicating no depression. Group 2 – *no depression, stable (low)* (N=838) – the largest group, had a slightly higher symptom level than group 1. Group 3 – *moderate depression, considerably decreasing* (N=38) – was small and had relatively high symptoms prior to retirement that decreased to no depression at the end of the period. Group 4 – *mild depression, decreasing* (N=326) – had symptoms of mild depression initially, which decreased to doubtful depression. Finally, group 5 – *moderate depression, stable* (N=62) – with symptoms of moderate depression remained on a similar level over time. As shown in figure 3, the points, which represent the average symptom score for each trajectory at each timepoint, are slightly above the fitted polynomial curves -1 year before retirement and below the lines in +1 year following retirement. This accords to the findings depicted in figure 2.

### Characteristics of the trajectories

Distribution of some demographic variables prior to retirement in the study sample as well as stratified by trajectory group are presented in table 1. Average retirement age was 66 years. There were significant differences in the distribution of sex, pre-retirement age, civil status and occupational position between the trajectory groups. In the group 3, 4 and 5 trajectories, there were, eg, larger proportions of women, singles and unskilled workers, compared to the proportions in the group 1 trajectory. Distribution of some health variables and the psychosocial working characteristics in the study sample and stratified by trajectory group are presented in table 2. There were significant differences in the distribution of physical inactivity, excessive drinking, smoking and cardiovascular disease and these factors were most common in the group 4 and 5 trajectories. In the group 3, 4 and 5 trajectories, there were, eg, larger proportions of individuals who perceived high demands, low decision authority, job strain, low social support, high efforts, low rewards, ERI and low procedural justice, compared to the proportions in the group 1 trajectory.

**Table 1:** Characteristics of the sample and of the depression trajectory groups in the SLOSH study, 2006–2018. Missing information: depressive symptoms change (N=74; 4.3%), civil status (N=10; 0.6%), occupational position (N=22; 1.3%). [SD=standard deviation.]

	Total		Trajectory groups										P-value ^a^
	
	N=1735		Group 1		Group 2		Group 3		Group 4		Group 5		
					
			No depression, stable (very low)		No depression, stable (low)		Moderate depression, considerably decreasing		Mild depression, decreasing		Moderate depression, stable		
					
	N	%	N	%	N	%	N	%	N	%	N	%	
Sex													
Men	805	46.6	269	57.1	361	43.1	14	36.8	134	41.1	27	43.6	<0.001
Women	930	53.6	202	42.9	477	56.9	24	63.2	192	58.9	35	56.5	
Civil status													
Single	348	20.2	72	15.4	166	19.9	12	32.4	80	24.8	18	29.5	<0.01
Married/cohabiting	1377	79.8	396	84.6	670	80.1	25	67.6	243	75.2	43	70.5	
Occupational position													
Unskilled workers	257	15.0	69	14.9	111	13.4	7	18.4	56	17.3	14	23.0	0.630
Skilled workers	268	15.7	74	16.0	136	16.4	3	7.9	47	14.6	8	13.1	
Assistant non-manual employees	272	15.9	67	14.5	126	15.2	5	13.2	62	19.2	12	19.7	
Intermediate non-manual employees	536	31.3	147	31.8	268	32.4	14	36.8	91	28.2	16	26.3	
Professionals/upper-level executives	336	19.6	93	20.1	167	20.2	7	18.4	58	18.0	11	18.0	
Self-employed	44	2.6	13	2.8	20	2.4	2	5.3	9	2.8	0	0.0	
		
	Mean	SD	Mean	SD	Mean	SD	Mean	SD	Mean	SD	Mean	SD	
		
Depressive symptom change ^b^	-1.5	4.3	-0.6	1.9	-1.4	3.9	-7.5	9.2	-2.6	5.5	-1.3	6.4	<0.001
Age at retirement (range 55–74 years)	65.9	1.9	65.9	2.0	66.0	1.8	65.3	2.8	65.7	2.0	66.0	2.0	<0.05

a Chi^2^ test for categorical variables and ANOVA for continuous variables.

b Change in the mean score of depressive symptoms between the wave prior to retirement (-1 year) and the wave in which retirement had taken place (+1 year).

**Table 2:** Health characteristics and psychosocial working characteristics of the sample and of the depression trajectory groups in the SLOSH study, 2006–2018. Missing information: physical inactivity (N=9; 0.5%), excessive drinking (N=49; 2.8%), smoking daily (N=9; 0.5%), cardiovascular disease (N=19; 1.1%), diabetes (N=23; 1.3%), job demands (N=23; 1.3%), decision authority (N=7; 0.4%), job strain (N=30; 1.7%), social support (N=47; 2.7%), efforts (N=215; 12.4%), rewards (N=255; 14.7%), effort–rewards imbalance (ERI) (N=265; 15.3%, efforts and rewards were not measured in SLOSH wave 2), procedural justice (N=124; 7.1%).

	Total		Trajectory groups										P-value ^a^
	
	N=1735		Group 1		Group 2		Group 3		Group 4		Group 5		
					
			No depression, stable (very low)		No depression, stable (low)		Moderate depression, considerably decreasing		Mild depression, decreasing		Moderate depression, stable		
					
	N	%	N	%	N	%	N	%	N	%	N	%	
Physical inactivity	310	18.0	66	14.1	146	17.5	8	21.6	72	22.3	18	29.0	<0.01
Excessive drinking	95	5.6	14	3.0	50	6.1	2	5.3	20	6.5	9	15.0	<0.01
Smoking (daily)	180	10.4	47	10.0	76	9.1	3	7.9	42	13.1	12	19.4	<0.05
Cardiovascular disease	170	9.9	24	5.1	81	9.8	1	2.6	50	15.7	14	23.0	<0.001
Diabetes	131	7.7	29	6.2	58	7.0	5	13.2	31	9.8	8	13.3	0.08
High job demands	668	39.0	125	26.8	327	39.5	22	57.9	163	51.1	31	50.8	<0.001
Low decision authority	717	41.5	169	36.0	350	41.9	21	55.3	152	46.9	31	51.7	<0.05
Job strain	262	15.4	40	8.6	121	14.7	14	36.8	71	22.3	16	26.7	<0.001
Low social support	674	39.9	135	29.2	319	38.9	25	65.8	163	52.9	32	53.3	<0.001
High efforts	538	35.4	129	30.9	251	34.3	18	52.9	117	41.6	23	41.8	<0.01
Low rewards	605	40.9	113	27.8	279	39.2	21	65.6	155	56.4	37	67.3	<0.001
ERI	722	49.1	160	39.5	340	48.1	24	75.0	164	60.5	34	61.8	<0.001
Low procedural justice	808	50.2	178	40.8	399	50.8	24	64.9	170	57.4	37	66.1	<0.001

a Chi^2^ test for categorical variables.

Associations between pre-retirement psychosocial working characteristics and trajectories of depressive symptoms

Table 3 shows the results from the multinomial logistic regression analyses to predict membership in the depression trajectories. In the crude models, perceiving high job demands, job strain, low social support, low rewards, ERI and low procedural justice prior retirement was associated with all trajectories of depressive symptoms with higher symptom level compared to the reference trajectory of group 1. If exposed to a “risky” level of psychosocial working characteristics, the risk estimates of belonging to (especially) the group 3 and 5 trajectories were large. On the other hand, perceiving low decision authority was not associated with a higher risk of belonging to the group 3 trajectory and high efforts were not significantly associated with the group 2 and 5 trajectories compared to the reference trajectory. After adjustments in models 1 and 2, all estimates remained statistically significant except for the group 2 and 5 trajectories associated with low decision authority. In general, the RR were slightly attenuated comparing the crude models with models 1. When comparing model 1 with model 2, some RR were attenuated, while others increased or remained unchanged (see table 3).

**Table 3:** Associations between psychosocial working characteristics, and the depression trajectories, presented as relative risk ratios (RR) and 95% confidence intervals (CI). [**Bold indicates statistically significant**].

Trajectories of depressive symptoms across retirement	Crude Model		Model 1 ^a^		Model 2 ^b^		N ^c^
		
	RR	95% CI	RR	95% CI	RR	95% CI	
Group 1: no depression, stable (very low)	1.00	(reference)	1.00	(reference)	1.00	(reference)	
High job demands							
Group 2: No depression, stable (low)	**1.94**	**1.50–2.50**	**1.88**	**1.45–2.45**	**1.88**	**1.44–2.45**	1622
Group 3: Moderate depression, considerably decreasing	**3.80**	**1.92–7.53**	**3.42**	**1.70–6.90**	**3.56**	**1.76–7.20**	
Group 4: Mild depression, decreasing	**3.03**	**2.22–4.14**	**2.96**	**2.15–4.07**	**2.93**	**2.12–4.05**	
Group 5: Moderate depression, stable	**2.89**	**1.64–5.10**	**2.95**	**1.65–5.26**	**2.85**	**1.58–5.14**	
Low decision authority							
Group 2: No depression, stable (low)	**1.29**	**1.01–1.64**	1.26	0.98–1.62	1.26	0.98–1.62	1633
Group 3: Moderate depression, considerably decreasing	1.50	0.77–2.95	1.43	0.71–2.89	1.43	0.70–2.90	
Group 4: Mild depression, decreasing	**1.53**	**1.14–2.06**	**1.39**	**1.02–1.90**	**1.41**	**1.03–1.93**	
Group 5: Moderate depression, stable	**1.71**	**0.97–3.00**	1.57	0.88–2.81	1.64	0.91–2.96	
Job strain							
Group 2: No depression, stable (low)	**1.96**	**1.32–2.91**	**1.88**	**1.26–2.81**	**1.84**	**1.23–2.75**	1616
Group 3: Moderate depression, considerably decreasing	**6.12**	**2.88–13.04**	**5.45**	**2.4811.95**	**5.52**	**2.50–12.18**	
Group 4: Mild depression, decreasing	**3.36**	**2.17–5.19**	**2.96**	**1.89–4.61**	**2.85**	**1.81–4.46**	
Group 5: Moderate depression, stable	**3.50**	**1.72–7.11**	**3.24**	**1.56–6.72**	**2.98**	**1.42–6.27**	
Low social support							
Group 2: No depression, stable (low)	**1.61**	**1.25–2.07**	**1.60**	**1.24–2.06**	**1.59**	**1.23–2.05**	1600
Group 3: Moderate depression, considerably decreasing	**4.50**	**2.22–9.11**	**4.28**	**2.08–8.80**	**4.44**	**2.15–9.14**	
Group 4: Mild depression, decreasing	**2.78**	**2.04–3.79**	**2.70**	**1.97–3.96**	**2.63**	**1.91–3.62**	
Group 5: Moderate depression, stable	**2.62**	**1.49–4.60**	**2.56**	**1.45–4.52**	**2.44**	**1.37–4.36**	
High efforts							
Group 2: No depression, stable (low)	1.16	0.90–1.51	1.09	0.83–1.42	1.08	0.83–1.42	1463
Group 3: Moderate depression, considerably decreasing	**2.51**	**1.24–5.09**	**2.07**	**1.014.25**	**2.12**	**1.03–4.36**	
Group 4: Mild depression, decreasing	**1.65**	**1.20–2.28**	**1.50**	**1.08–2.08**	**1.50**	**1.07–2.09**	
Group 5: Moderate depression, stable	1.56	0.86–2.84	1.48	0.80–2.71	1.38	0.74–2.57	
Low rewards							
Group 2: No depression, stable (low)	**1.72**	**1.31–2.24**	**1.67**	**1.27–2.20**	**1.62**	**1.23–2.13**	1429
Group 3: Moderate depression, considerably decreasing	**5.02**	**2.34–10.76**	**4.64**	**2.14–10.06**	**4.65**	**2.14–10.08**	
Group 4: Mild depression, decreasing	**3.48**	**2.51–4.84**	**3.31**	**2.37–4.62**	**3.15**	**2.25–4.41**	
Group 5: Moderate depression, stable	**5.26**	**2.82–9.81**	**5.10**	**2.72–9.56**	**4.62**	**2.44–8.75**	
Effort–reward imbalance							
Group 2: No depression, stable (low)	**1.46**	**1.13–1.88**	**1.38**	**1.06–1.78**	**1.38**	**1.06–1.79**	1419
Group 3: Moderate depression, considerably decreasing	**4.72**	**2.07–10.77**	**3.88**	**1.68–8.99**	**4.00**	**1.73–9.27**	
Group 4: Mild depression, decreasing	**2.49**	**1.81–3.44**	**2.28**	**1.64–3.18**	**2.26**	**1.62–3.16**	
Group 5: Moderate depression, stable	**2.25**	**1.24–4.07**	**2.19**	**1.19–4.02**	**2.09**	**1.12–3.88**	
Low procedural justice							
Group 2: No depression, stable (low)	**1.48**	**1.16–1.89**	**1.46**	**1.14–1.87**	**1.46**	**1.14–1.87**	1535
Group 3: Moderate depression, considerably decreasing	**2.50**	**1.23–5.07**	**2.46**	**1.18–5.11**	**2.51**	**1.20–5.26**	
Group 4: Mild depression, decreasing	**1.82**	**1.34–2.47**	**1.74**	**1.27–2.37**	**1.72**	**1.26–2.36**	
Group 5: Moderate depression, stable	**2.99**	**1.63–5.50**	**2.91**	**1.57–5.37**	**2.86**	**1.53–5.34**	

a Adjusted for sex, age, occupational position, civil status.

b Model 1 + physical inactivity, excessive drinking, smoking, cardiovascular disease, diabetes.

c Number of individuals in the crude model, Model 1 and Model 2, for each exposure variable.

## Discussion

### Main findings

Our results seem to support a beneficial effect of retirement, albeit quite modest, in terms of depressive symptoms. This is in line with a review concluding that retirement has beneficial effects on mental health (3), and other studies observing decreasing depressive symptoms in relation to retirement (14). Our results were also in line with those by Fleischmann et al (16), which showed that mental health improves already before retirement. However, we adopted a different analytic strategy than many previous studies on this topic and supported a heterogenous development of depressive symptoms across retirement (11, 12).

We further found that perceptions of job demands, job strain, workplace social support, rewards, ERI and procedural justice were associated with all the trajectories of depressive symptoms, while perceptions of decision authority and work efforts were only related to some of the trajectories. Interestingly, increased risks of belonging to even the no depression, stable (low) (group 2) trajectory for those with worse working characteristics when compared to the lowest reference trajectory were observed, indicating that poor working conditions may be associated with worse mental health, even in individuals with a low symptom level. In a previous paper, we similarly found that high demands and low social support predicted trajectories with higher levels of depressive symptoms while in working life (35). The fact that most of the psychosocial working characteristics were associated with a higher probability of belonging to the moderate depression, considerably decreasing (group 3) trajectory, may indicate that the retirement-related relief from exposure to these work stressors could have a beneficial effect on depressive symptoms. Fleischmann et al (16) also found support for such a relief where positive changes in mental health were more explicit for those retiring from poorer working conditions including high job demands, lower social support, or lower decision authority. This was in line with our findings except for the results regarding decision authority. Westerlund et al (13) similarly found that high (physical and psychological) job demands were associated with a more pronounced retirement-related improvement regarding self-rated health, suggesting that perceived health problems are relieved by retirement among older workers with poor working conditions. However, since the group 3 trajectory included so few individuals, more evidence is needed to substantiate this finding. Several psychosocial working characteristics were on the other hand, also associated with the moderate depression, stable (group 5) trajectory with highest depressive symptom scores, which seem to support previous findings suggesting that job demands or psychosocial stress at work (operationalized with the job demand–control and ERI models) during midlife were associated with post-retirement mental health (18, 19). This implies that job demands/efforts may have long-term effects on mental health, and our results also suggest that this may be true for workplace social support, rewards and organizational justice.

### Strengths and limitations

This study contributed to the field by addressing a need for longitudinal studies regarding potentially influencing factors like work characteristics in relation to mental health effects of retirement (3). In contrast to a similar study treating all retirees as one group using piecewise trajectories (16), we applied group-based trajectory modelling to identify subgroups of retirees with different patterns of depressive symptoms across retirement. We thereby considered that retirees do not constitute an homogenous group (11, 12, 40), with individuals possibly experiencing deteriorating, improved or stable mental health. This study thus contributes to the current literature by supporting that mental health across retirement is heterogenous and that factors like work characteristics may play a role. Furthermore, we analyzed a rather large sample, approximately representative of the Swedish working population, thereby increasing the study’s generalizability. Many previous studies have been relying on cross-sectional designs or only two waves, and moreover, few previous studies have used designs that are effective in terms of catching the effect of retirement on health and vice versa (8). Latent class growth analysis may also be suitable to capture different patterns such as recurrence and remission in depressive symptoms, which are known to exist in depression and may have different consequences (41). Our associations were generally robust when controlling for sex, age, occupational position, civil status, health risk behaviors, cardiovascular disease and diabetes, indicating that these factors did not explain the associations to a large extent. However, we cannot exclude that other unmeasured factors unrelated to work co-occurred with the retirement transition and thus may explain the decreased levels of depressive symptoms.

Some limitations include that only small proportions of the retirees followed the patterns of symptoms in the group 3 (2.2%) and 5 (3.6%) trajectories. This reduces the reliability of the findings regarding patterns of symptom development and leads to wide CI for the risk estimates of the relationship between work characteristics and these trajectories. We used dichotomous exposure variables and thereby lost some information. We only included individuals who had responded to several SLOSH questionnaires, who are possibly healthier and thus depressive symptom levels could be underestimated. A limited exposure contrast is also possible, which may result in underestimation of the associations.

When investigating the trajectories visually, there seemed to be a tendency for the symptom level to slightly increase around 9–11 years following retirement. It should be noted that there were relatively few observations so many years before and after retirement and that the selection of the shapes of trajectories based on polynomial functions of time/age is known to generate patterns unsupported by the data, such as uplifts at each end of the time axis (42). These patterns should therefore be interpreted with caution. It should also be acknowledged that the trajectories may not fully capture individual change, it can be difficult to identify trajectories with different shapes (43) and that we cannot draw causal conclusions. However, if causal, these results stress the value of workplace interventions targeting these types of work characteristics for healthy and active aging. Improved working environment may also enable increased retirement age.

### Concluding remarks

Our findings generally indicated a modest, yet positive effect of retirement on depressive symptoms in a sample of Swedish retirees, with variation between groups and a small group showing a clear improvement. Furthermore, a relief from poor psychosocial working characteristics seemed to be associated with a more significant improvement. However, poor working characteristics were also associated with persistent symptoms suggesting a long-term effect of psychosocial working characteristics on depressive symptoms.

## Supplementary material

Supplementary material
