# Supplementary material for: Psychosocial working characteristics before retirement and depressive symptoms across the retirement transition: a longitudinal latent class analysis
Source: Scand J Work Environ Health. 2020 Feb 24;46(5):488–97. doi: 10.5271/sjweh.3889 (PMC7737805; doi:10.5271/sjweh.3889)
Supplement: Supplementary material [file SJWEH-46-488-S001.pdf]

Psychosocial working characteristics before retirement and depressive symptoms across the retirement transition: a longitudinal latent class analysis [1]

by Julia K Åhlin, PhD,[2] Paraskevi Peristera, PhD, Hugo Westerlund, PhD, Linda L Magnusson Hanson, PhD

1. *Supplementary material*
2. *Correspondence to: Julia Åhlin, The Division for Epidemiology, Stress Research Institute, Department of Psychology, Stockholm University, Stockholm SE-106 91, Sweden. [E-mail: julia.ahlin@su.se]*

Supplementary table S1. Sensitivity analysis comparing the characteristics of the 621 excluded individuals without data on depressive symptoms in at least four waves and the 1735 included individuals, in the SLOSH study between 2006 and 2018.

		Excluded	Included	Difference
		**N=621[a]**	**N=1735**	**p[b]**
**Sex**	Men %	47.5	46.6	0.635
	Women %	52.5	53.6	
**Depressive symptom change [c]**		-1.3	-1.5	0.170
**Age at retirement** Mean (Sd)		65.9 (2.1)	65.9 (1.9)	0.706
**Civil status**	Single %	20.7	20.2	0.760
	Married/cohabiting %	79.3	79.8	
**Occupational position**	Unskilled workers %	18.0	15.0	0.202
	Skilled workers %	16.7	15.7	
	Assistant non-manual employees %	14.5	15.9	
	Intermediate non-manual employees %	29.1	31.3	
	Professionals/Upper-level executives %	20.4	19.6	
	Self-employed %	1.3	2.6	
**Health characteristics**	Physical inactivity %	19.0	18.0	0.580
	Excessive drinking %	5.9	5.6	0.823
	Smoking (daily) %	10.6	10.4	0.884
	Cardiovascular disease %	10.3	9.9	0.801
	Diabetes %	7.1	7.7	0.669
**Psychosocial work stressors**	High job demands %	38.7	39.0	0.892
	Low decision authority %	46.0	41.5	0.165
	Job strain %	15.2	15.4	0.900
	Low social support %	43.4	39.9	0.143
	High efforts %	36.2	35.4	0.734
	Low rewards %	44.6	40.9	0.132
	ERI %	44.4	49.1	0.063
	Low procedural justice %	47.6	50.2	0.326

[a] Missing information for excluded individuals: sex - , depressive symptoms change (n=36, 5.8%), age at retirement - , civil status (n=9, 1.4%), occupational position (n=22, 3.5%), physical inactivity (n=4, 0.6%), excessive drinking (n=26, 4.2%), smoking daily (n=10, 1.6%), cardiovascular disease (n=17, 2.7%), diabetes (n=17, 2.7%), job demands (n=19, 3.1%), decision authority (n=8, 1.3%), job strain (n=27, 4.3%), social support (n=45, 7.2%), effort (n=63, 10.1%), reward (n=90, 14.5%), ERI (n=94, 15.1%), procedural justice (n=142, 22.9%). [b] Chi^2 test for categorical variables and T-test for continuous variables. [c] Change in the mean score of depressive symptoms comparing the wave prior to retirement (-1 year) and the wave in which retirement had taken place (+1 year).

Supplementary description of variables.

Exposure variables

Job demands, decision authority and social support

Four items (working fast, too much effort, enough time and conflicting demands) were used to calculate the job demand score (mean $\alpha = 0.69$) and two items (deciding what to do at work, deciding how to do your work) were used for the subdimension decision authority of job control (mean $\alpha = 0.75$), ranging from "Never/almost never" 1) to "Often" 4) (1). Five items (calm and pleasant atmosphere, good spirit of unity, colleagues are there for me, people understand a bad day, get on well with my supervisors) were used for the workplace social support score (mean $\alpha = 0.84$), ranging from "Strongly disagree" 1) to "Strongly agree" 4). The median values across waves for job demands, decision authority and social support were 2.5, 3.0-3.5 and 3.0-3.1 respectively.

Efforts, rewards and effort-reward imbalance

Three items were used for the work efforts score (time pressure due to work load, job become more demanding and workload increased) (mean $\alpha = 0.78$), while rewards were assessed using seven items (lack acknowledgement supervisor, poor promotion prospects, experience(d) undesirable change, job security poor, not receive respect/prestige, work prospects adequate, salary/income adequate) (mean $\alpha = 0.71$), ranging from "Strongly disagree" 1) to "Strongly agree" 4). We also calculated the effort-reward imbalance (ERI) ratio by dividing efforts by rewards (with rewards multiplied by a correction factor of $3/7 \approx 0.43$ to adjust for unequal number of items of the two scales). A ratio of >1 indicated a high level of effort that is not met by the rewards received or expected, while a ratio <1 indicated a favourable condition of relatively low efforts in relation to rewards (2). The

median values across waves for effort, reward and effort reward imbalance were 8-11, 18-21 and 1.0-1.2 respectively.

Procedural justice

Seven items were used to assess procedural justice (decisions taken correctly, bad decisions revoked/changed, all sides affected represented, decisions taken consistently, everyone give their opinion, feedback provided and people informed, possible obtain details underlying decision) (mean α = 0.91), with response options ranging from "Strongly agree" 1) to "Strongly disagree" 5). The median values across waves for procedural justice were 3.3-3.7.

Covariates

Demographic characteristics included sex (men/women), age at retirement, civil status (single, or married/cohabiting) and occupational position. Occupational position was used as a measure to indicate socioeconomic position and was based on the Swedish socioeconomic classification (SEI) carried out by Statistics Sweden (3) and consisted of six categories (unskilled workers, skilled workers, assistant non-manual employees, intermediate non-manual employees, professionals/upper-level executives or self-employed).

Health characteristics included three measures of health risk behaviours, as well as two common chronic diseases, namely cardiovascular disease and diabetes. Physical inactivity was measured by the question *How much exercise do you get? Include any walking or cycling you do to work*. and those who responded that they never exercise or who responded that they do not exercise very much were defined as physically inactive. Excessive alcohol consumption was measured by the Alcohol Use Disorders Identification Test (AUDIT) in wave one and two (4). Two questions regarding frequency of alcohol consumption (*How often do you drink?*) and amount of alcohol (*How many "glasses" (see below) do you drink on a typical day when you drink alcohol?*) were combined to create a measure of excessive alcohol consumption with a cut-off of 14 units or more/week for women and 21 units or more/week for men. In wave three through five, excessive alcohol consumption was measured by the CAGE questionnaire, a mnemonic for attempts to Cut back on drinking, being Annoyed at criticisms about drinking, feeling Guilty about drinking, and using alcohol as an Eye opener (5). Four yes/no questions were used (*Have you ever felt you should cut down on your drinking?, Have people annoyed you by criticising you drinking?, Have you at several times had a drink first thing in the morning to steady your nerves or get rid of a hangover? and Do you feel bad or guilty about your drinking?*). Individuals were defined as having an excessive

alcohol consumption if they answered “yes” to at least one of these questions. One questions was used to measure smoking: *Do you currently smoke?* and those answering “Yes every day” were defined as smoking (daily).

Cardiovascular disease and diabetes were measured by the question *Has a doctor told you that you have… a heart disease* or *diabetes?* (yes/no) in wave one. In wave two through five the question was expanded to *Have you or have you had one or more of the following protracted and/or serious illnesses or complaints during the last 2 years? (Cardiovascular disease/diabetes)* with the answer options: “No”, “Yes, but it does not influence my life”, “Yes, it influences my life a little” or “Yes, it influences my life to a great deal”. All answer options including yes were defined as having cardiovascular disease or diabetes.

Outcome variable

Depressive symptoms

The six items measured to assess depressive symptoms (*feeling blue, feeling no interest in things, feeling lethargic or low in energy, worrying too much about things, blaming oneself for things* and *feeling everything is an effort*) (mean α = 0.91) represent core symptoms, selected based on principles of clinical validity (6). The SCL-CD_6 scale has been validated and found to have good psychometric properties, including adequate construct validity, high unidimensionality and predictive of hospitalization and antidepressant medication (7).

References:

1. Chungkham HS, Ingre M, Karasek R, Westerlund H, Theorell T. Factor Structure and Longitudinal Measurement Invariance of the Demand Control Support Model: An Evidence from the Swedish Longitudinal Occupational Survey of Health (SLOSH). PLoS One. 2013;8(8).
2. Siegrist J, Starke S, Chandola T, Godin I, Marmot M, Niedhammer I, et al. The measurement of effort-reward imbalance at work: European comparisons. Soc Sci Med. 2004;58(8):1483-99. doi: 10.1016/S0277-9536(03)00351-4.
3. Statistics Sweden. Swedish socio-economic classification (SEI). Stockholm: Statistiska Centralbyrån (SCB) 1984.
4. Saunders JB, Aasland OG, Babor TF, de la Fuente JR, Grant M. Development of the Alcohol Use Disorders Identification Test (AUDIT): WHO Collaborative Project on Early Detection of Persons with Harmful Alcohol Consumption--II. Addiction. 1993;88(6):791-804.
5. Bush B, Shaw S, Cleary P, Delbanco TL, Aronson MD. Screening for Alcohol Abuse Using the CAGE Questionnaire. Am J Med. 1987;82(2):231-5. doi: 10.1016/0002-9343(87)90061-1.
6. Bech P. The use of rating scales in affective disorders. European Psychiatric Review. 2008;1:14-8.

7. Magnusson Hanson LL, Westerlund H, Leineweber C, Rugulies R, Osika W, Theorell T, et al. The Symptom Checklist-core depression (SCL-CD6) scale: psychometric properties of a brief six item scale for the assessment of depression. Scand J Public Health. 2014;42(1):82-8. doi: 10.1177/1403494813500591.
